# Th17 cells are associated with protection from ventilator associated pneumonia

**DOI:** 10.1371/journal.pone.0182966

**Published:** 2017-08-14

**Authors:** Marika Orlov, Victoria Dmyterko, Mark M. Wurfel, Carmen Mikacenic

**Affiliations:** 1 Department of Medicine, University of Washington, Seattle, Washington, United States of America; 2 Department of Medicine, Division of Pulmonary and Critical Care Medicine, University of Washington, Seattle, Washington, United States of America; Hospital for Sick Children, CANADA

## Abstract

**Background:**

CD4+ T-helper 17 (Th17) cells and Interleukin (IL)-17A play an important role in clearing pathogens in mouse models of pneumonia. We hypothesized that numbers of Th17 cells and levels of IL-17A are associated with risk for nosocomial pneumonia in humans.

**Methods:**

We collected bronchoalveolar lavage (BAL) fluid from mechanically ventilated (n = 25) patients undergoing quantitative bacterial culture to evaluate for ventilator associated pneumonia (VAP). We identified Th17 cells by positive selection of CD4+ cells, stimulation with ionomycin and PMA, then staining for CD4, CD45, CCR6, IL-17A, and IFN-γ followed by flow cytometric analysis (n = 21). We measured inflammatory cytokine levels, including IL-17A, in BAL fluid by immunoassay.

**Results:**

VAP was detected in 13 of the 25 subjects. We identified a decreased percentage of IL-17A producing Th17 cells in BAL fluid from patients with VAP compared to those without (p = 0.02). However, we found no significant difference in levels of IL-17A in patients with VAP compared to those without (p = 0.07). Interestingly, IL-17A levels did not correlate with Th17 cell numbers. IL-17A levels did show strong positive correlations with alveolar neutrophil numbers and total protein levels.

**Conclusions:**

Th17 cells are found at lower percentages in BAL fluid from mechanically ventilated patients with VAP and IL-17A levels correlated with Th17 cell percentages in non-VAP subjects, but not those with VAP. These findings suggest that Th17 cells may be protective against development of nosocomial pneumonia in patients receiving mechanical ventilation and that alveolar IL-17A in VAP may be derived from sources other than alveolar Th17 cells.

## Introduction

Hospital-acquired infections are common and are associated with increased morbidity and mortality. Hospital acquired pneumonias (HAPs) are the most common form of nosocomial infection and are associated with the highest mortality (30–50% of all hospital acquired infections)(1). Ventilator associated pneumonia (VAP), a form of HAP acquired after at least 48 hours of intubation and mechanical ventilation, is associated with the worst outcomes, with intensive care unit (ICU) mortality rates ranging from 24–75%[1]. Our incomplete understanding of the immunologic mechanisms leading to development of VAP is an obstacle to the development of strategies for preventing VAP.

One possible mechanism contributing to the risk of VAP in critically ill patients is”immunoparalysis,” also known as the compensatory anti-inflammatory response syndrome (CARS), which may result in increased susceptibility to secondary infections [2–4]. CARS is thought to follow the pro-inflammatory “cytokine storm” seen in early sepsis and is characterized by apoptosis of immune cells, impaired lymphocyte and phagocyte function, and a shift from a Th1 to a Th2 immune phenotype. It is unclear to what extent impaired immune function might participate in altering the risk for lower respiratory tract infections in the setting of mechanical ventilation.

Studies in mouse models have shown that T-helper 17 (Th17) cells, which are a subset of CD4+ T helper cells, play an important role in host defense and clearance of bacterial and fungal pathogens from the lung [5–8]. Th17 cells differentiate in the setting of a pro-inflammatory cytokine milieu and secrete cytokines such as IL-17A, IL-17F, and IL-22, which are also known to have pro-inflammatory properties. IL-17A has been shown to confer protection in murine pulmonary infections against extracellular and intracellular bacterial pathogens such as *Klebseilla pneumonia* and *Mycoplasma pneumonia*, respectively [5]. The mechanisms involved in protection include increased neutrophil recruitment, increased anti-microbial peptide secretion as well as increased expression of cell adhesion molecules such as ICAM-1 [5]. However, in murine models of lung injury, increased levels of IL-17A correlate with increased alveolar leak and worse outcomes [9]. While Th17 cells are thought to be the main source of IL-17A, there are many other cells types that produce IL-17A such as CD8+ T-cells [10], NK T-cells [11], γδ-T cells [12], innate lymphoid cells [13], and neutrophils [14,15]. The relationship between Th17 cells and production of IL-17A protein in patients at risk for development of nosocomial pneumonia is unknown.

The role that Th17 cells and IL-17A play in human lung infections and lung disease is unclear. One study of community acquired pneumonia (CAP) showed an increased percentage of IL-17A/IL-22 double positive cells in the periphery and bronchoalveolar lavage (BAL) of patients with CAP compared to healthy controls [16]. We have shown that levels of IL-17A are elevated in patients with ARDS, many of whom had pneumonia [17]. In cystic fibrosis, levels of alveolar IL-17A producing CD4+ cells are increased [18]. Levels of IL-17A are also elevated in patients with severe COPD and asthma compared to mild and moderate disease [19,20]. In this study we examine the relationship between alveolar Th17 cells, alveolar IL-17A and the presence of ventilator associated pneumonia.

## Methods

### Subjects

Specimens were collected from intubated and mechanically ventilated patients at Harborview Medical Center undergoing diagnostic bronchoscopies to evaluate for ventilator associated pneumonia. This study was approved by the University of Washington Human Subjects Committee and informed consent was obtained through the legal next of kin. Inclusion criteria included mechanical ventilation >48 hours and a clinical suspicion for ventilator associated pneumonia based on the presence of fever, increased respiratory secretions or new alveolar opacity on chest radiography. VAP was defined according to the new IDSA guidelines for VAP (>10,000 CFUs in BAL fluid or >1,000 CFUs if using a protected specimen brush, n = 10) or if the quantitative culture did not meet CFU thresholds but grew a highly pathogenic species in the setting of high clinical suspicion (n = 3, S1 Table).

### Cell isolation

The BAL sample was filtered through a 70μm cell strainer and then centrifuged at 1200rpm for 5 min. The supernatant was removed and stored at -80°C to be used as “BAL fluid” for cytokine measurements (below). The pellet was mixed with RBC Lysis buffer (Miltenyi), gently vortexed and incubated at room temperature (RT) for 15 minutes. The cells were then spun at 1200rpm for 5 min and supernatant was aspirated. Cell pellet was resuspended in 10mL phosphate buffered saline (PBS) with 2% fetal bovine serum (FBS). Cells were counted and cytospin slides were made for counting cell types (see below). The remainder of the cells were centrifuged at 1200rpm for 5 min and supernatants was aspirated. The cells were resuspended in 7% dimethyl sulfoxide(DMSO)/FBS solution and transferred to 1mL cryovials (max 20x10^6 cells per vial). The cryovials were frozen overnight at -80°C with gradual cooling (1 degree C/min) and then placed into liquid nitrogen the following morning.

### Cytokine quantification

BAL fluid was stored at -80°C as above. All BAL fluid was thawed at once and cytokine concentration determined using a multiplex chemiluminescent immunoassay per the manufacturer’s protocol (IL-17A singleplex and Pro-inflammatory cytokines multiplex; Mesoscale Discovery). The lower limits of detection for the assay were as follows: TNF-α: 0.51 pg/mL, IFN-γ: 1.7 pg/mL, IL-1β: 0.15 pg/mL, IL-8: 0.15 pg/mL, IL-12p70: 0.69 pg/mL, IL-6: 0.33 pg/mL, IL-10: 0.14 pg/mL, and IL-17A: 2.07 pg/mL.

### Phenotyping airway lumen cells byFlow cytometry

Bronchoalveolar lavage cells were thawed and CD4 cells were isolated by positive selection per manufacturer’s protocol using the MACS^®^ system (Miltenyi, Auburn, CA). Cells eluted from the column were resuspended in 200uL of RPMI complete media (containing 1% L-glutamine, 1% pen-strep, 1% sodium-pyruvate, and 10% human AB serum) and plated in a 96 well u-bottom plate. The cells were then stimulated with PMA and Ionomycin at 500ng/mL (each) for 1 hour and then Brefeldin A and Monensin were added to the culture media at 1:1,000 dilution (Biolegend, 1000x stock) for an additional 3 hours. Once the stimulation was complete, cells were washed in PBS, spun and labeled first with a fixable Live/Dead cell marker (eFlor 780, eBiosciences) at 1:6,600 dilution in PBS for 10 minutes. After this treatment the stimulated cells were stained for extracellular markers: CD45 FITC, CD4 PerCP Cy5.5, and CCR6 PE-Cy7 (BioLegend) for 30 minutes in FACS buffer (PBS, 1% Bovine Serum Albumin to block non-specific binding, and 0.1% sodium azide) Cells were then fixed and permeabilized using FoxP3 staining buffer reagents per manufacturer’s instructions (eBiosciences). Once fixed and permeabilized, the cells were stained with the following intracellular markers: IL-17A BV605 (BioLegend) and IFN-γ-V450 (BD Biosciences) for 30 minutes. The cells were washed 1 additional time and resuspended in FACS buffer. All flow cytometry was acquired on the FACSCanto II (BD Biosciences) the same day as the cell isolation and staining.

### Neutrophil quantification

Cytospins were prepared by diluting 50,000 BAL cells (isolated as above) in 500uL of PBS, adding 250μL of cells to the slide and allowing them to dry at room temperature. The slides were stained per manufacturer’s instructions using Hema 3 stain (Fisher Scientific). Different cell populations were manually counted using light microscopy. 100 cells were counted per high power field and 2 high power fields were counted per slide.

### Protein quantification

BAL fluid was isolated and frozen as above. The fluid was thawed and protein quantified by bicinchoninic acid protein assay per the manufacturer’s instructions (BCA Protein assay, Thermo Fisher).

### Statistical analysis

When there were 2 populations being compared, a 2-tailed T-test was used. When there were more than 2 populations being compared, ANOVA analysis was used. All flow cytometric data was analyzed after calculation of the number of Th17 cells as a percentage of all the live CD4+ cells isolated (Th17 cells and IFN-γ+ cells). All graphs were made using GraphPad Prism. We have made our data available in the supplementary material (S1 Dataset).

## Results

We enrolled 25 patients undergoing diagnostic bronchoscopy to evaluate for VAP from the medical, surgical and neurologic/neurosurgical Intensive Care Units (ICUs) at Harborview Medical Center, Seattle, WA. VAP was identified in 13 (52%) patients (Table 1). The study subjects were predominantly Caucasian (76%) and male (96%). Trauma was the admitting diagnosis for the majority of the subjects (56%). Notably, there was a higher prevalence of ARDS in subjects with VAP compared to those with no VAP (69% vs 17%, respectively), however the APACHE III score at the time of Harborview ICU admission was no different between the VAP and no VAP groups (63 vs 61). Timing of BAL differed somewhat between the patients with and without VAP (10 days vs 6 days). Nearly all (n = 23) of the patients experienced symptoms concerning for VAP less than 24 hours prior to the bronchoscopy.

**Table 1:** Demographic data.

	VAP^a^ Status	
	Yes (n = 13)	No (n = 12)
**Age (mean ± SD)**	51 ± 14	49 ± 18
**Caucasian (n, %)**	10 (77%)	9 (75%)
**Male (n, %)**	13 (100%)	11 (92%)
**Acute Respiratory Distress Syndrome (ARDS) (n, %)**	9 (69%)	2 (17%)
**PaO**_**2**_**:FiO**_**2**_ **Ratio (mean ± SD)**	205 ± 59	354 ± 104
**Ventilator Days Prior to Bronchoscopy (mean ± SD)**	10 ± 7	6 ± 4
**APACHE III Score (mean ± SD)** ^b^	63 ± 23	61 ± 19
**Days in ICU prior to Bronchoscopy (median, range)**	4 (1–16)	8 (2–25)
**Admit Diagnosis**		
Trauma	9	5
Medical	1	6
Cerebrovascular	3	1

^a^VAP = ventilator associated pneumonia

^b^Acute Physiology and Chronic Health Evaluation III

### Reduced proportion of Th17 cells associated with VAP and relationship to IL-17A levels

**Fig 1:**
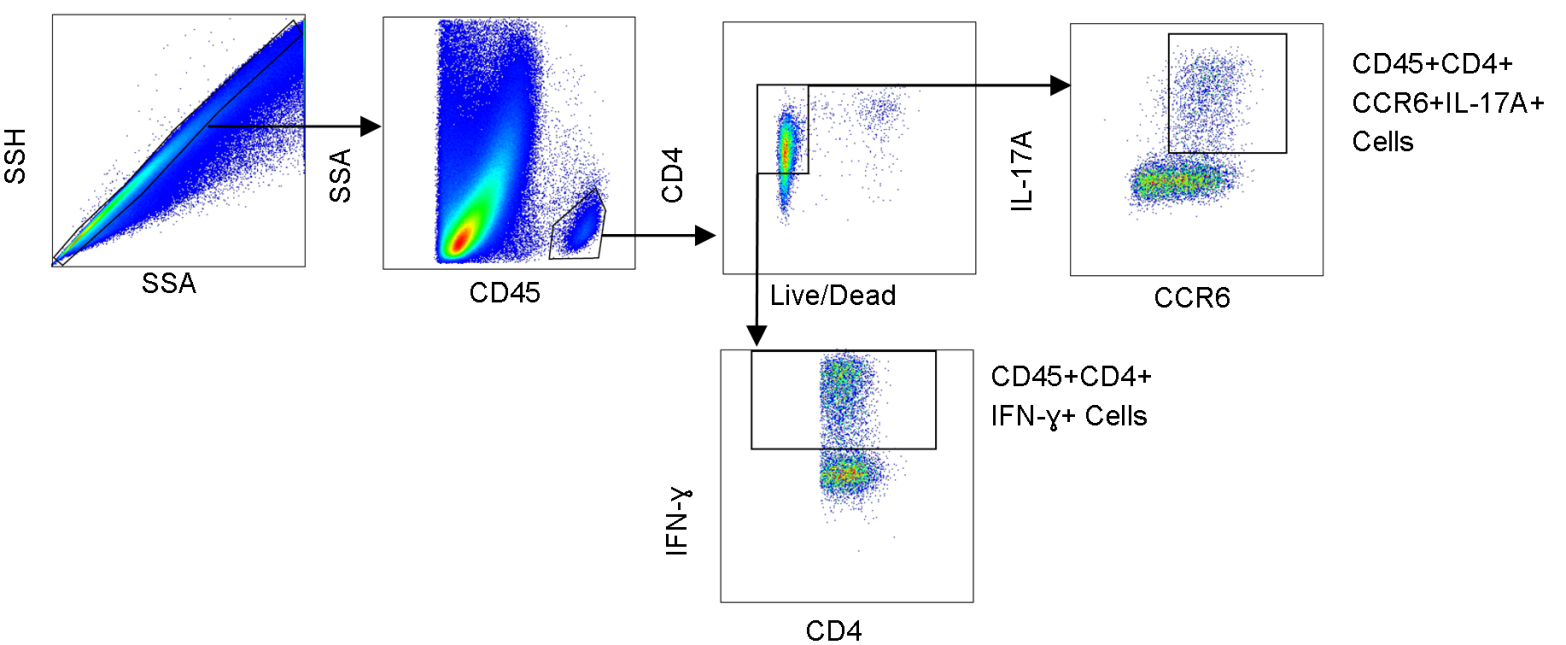
Representative flow cytometry gating strategy. Alveolar cells were surface stained for CD45, CD4, CCR6 and then intracellularly stained for IL-17A and Interferon-gamma (IFN-ɣ). We gated on single cells, CD45+ cells, live CD4+ T cells, and then for either CCR6+IL17A+ cells or total IFN-ɣ producing cells. SSH: Side Scatter Height, SSA: Side Scatter Area.

**Fig 2:**
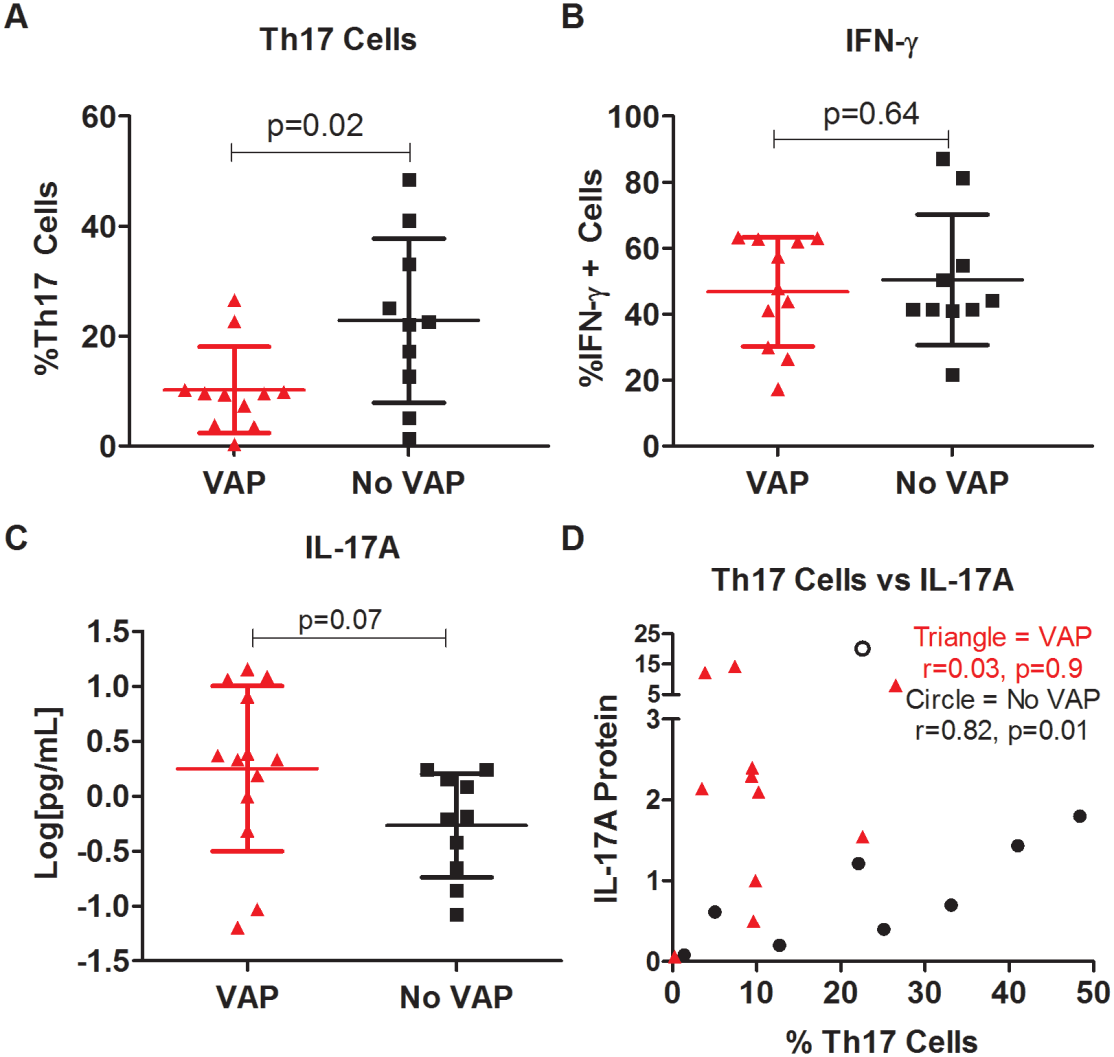
Percentage of Th17 cells reduced in the setting of ventilator associated pneumonia. Cells were isolated from bronchoalveolar lavage fluid, stained, and percentages of Th17 cells and CD4+ IFN-γ+ positive cells were obtained using flow cytometry. A: Percentage of Th17 cells (CCR6+IL-17A+) of the total CD4+ population in the BAL of patients with VAP or without VAP. B. Percentage of IFN-γ+ cells of the total CD4+ population. C. IL-17A protein concentrations measured by immunoassay in the BAL of subjects with and without VAP. D. We compared the relationship between numbers of Th17 cells (x-axis) and amount of IL-17A protein (y-axis) in subjects with VAP (triangles) and without VAP (circles).

We determined the proportion of Th17 cells (CD45+CD4+CCR6+IL-17A+) and IFN-γ+ Th1 cells (CD45+CD4+IFN-γ+) in the alveolar fluid as a percentage of the total CD4+ population. Our gating strategy is included in Fig 1. In subjects with VAP, we saw a significantly lower percentage of Th17 cells compared to the no VAP group (p = 0.02, Fig 2A). There was no significant difference in the percentage of IFN-γ producing CD4 cells (CD45+CD4+IFN-γ+) between the two groups (p = 0.64, Fig 2B). We then quantified the amount of IL-17A protein in the BAL fluid and found that there was a numeric trend towards higher IL-17A in patients with VAP, however that difference was not statistically significant (p = 0.07, Fig 2C).

We next tested for a relationship between Th17 cell numbers and IL-17A protein levels in the VAP and no VAP groups. In the absence of VAP, there is a significant correlation between Th17 cell numbers and IL-17A protein levels (r = 0.82, p = 0.01, Fig 2D circles). However, in the presence of VAP, this correlation is no longer present (r = 0.03, p = 0.9, Fig 2D triangles).

### Increased pro-inflammatory cytokine levels in BAL of VAP patients

**Fig 3:**
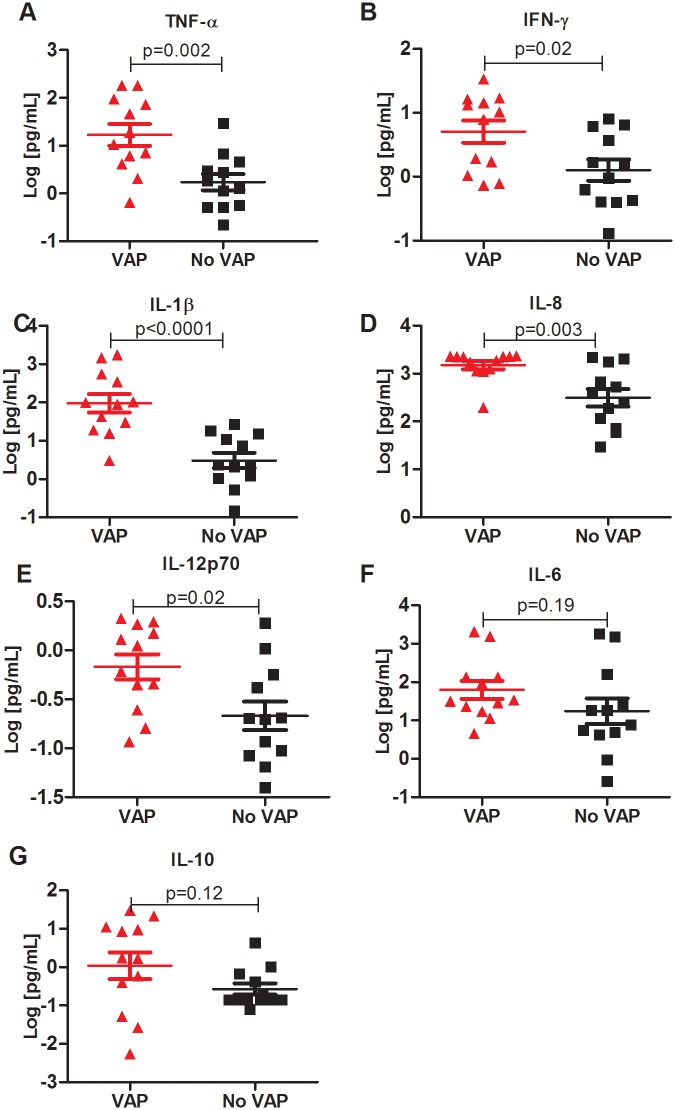
Increased levels of pro-inflammatory cytokines in bronchoalveolar lavage samples of patients with ventilator associated pneumonia. Bronchoalveolar lavage fluid was isolated from patients with and without ventilator associated pneumonia (VAP) and run on a multi-plex immuno assay to measure cytokine protein levels. Subjects with VAP (red triangles) are compared to those without VAP (black circles). All y-values are the log of the concentration in pg/mL. p-values were measured using a 2-tailed T-test. For most pro-inflammatory cytokines, such as A: TNF-α, B: IFN-γ, C: IL-1β, and E: IL-12p 70, and for the chemokine IL-8 (D), there was increased expression in the VAP samples. For IL-6 (F) and IL-10 (G), there was no significant difference between the two groups.

We then measured cytokine levels in the BAL fluid obtained from patients with and without VAP. We saw an increase in the levels of most pro-inflammatory cytokines in the BAL of VAP patients compared to those with no VAP (Fig 3). Cytokines such as TNF-α (p = 0.002 Fig 3A), IFN-γ (p = 0.02, Fig 3B), IL-1β (p<0.001, Fig 3C), and IL-12p70 (p = 0.02, Fig 3E) were significantly elevated in patients with VAP, as was the chemokine IL-8 (p = 0.003, Fig 3D). However levels of other pro-inflammatory cytokines such as IL-6 (p = 0.19, Fig 3F) were no different between the two groups. IL-10 was no different between the VAP and no VAP groups (p = 0.12, Fig 3G).

### IL-17A protein levels associated with increased protein levels in BAL and increased neutrophil recruitment

**Fig 4:**
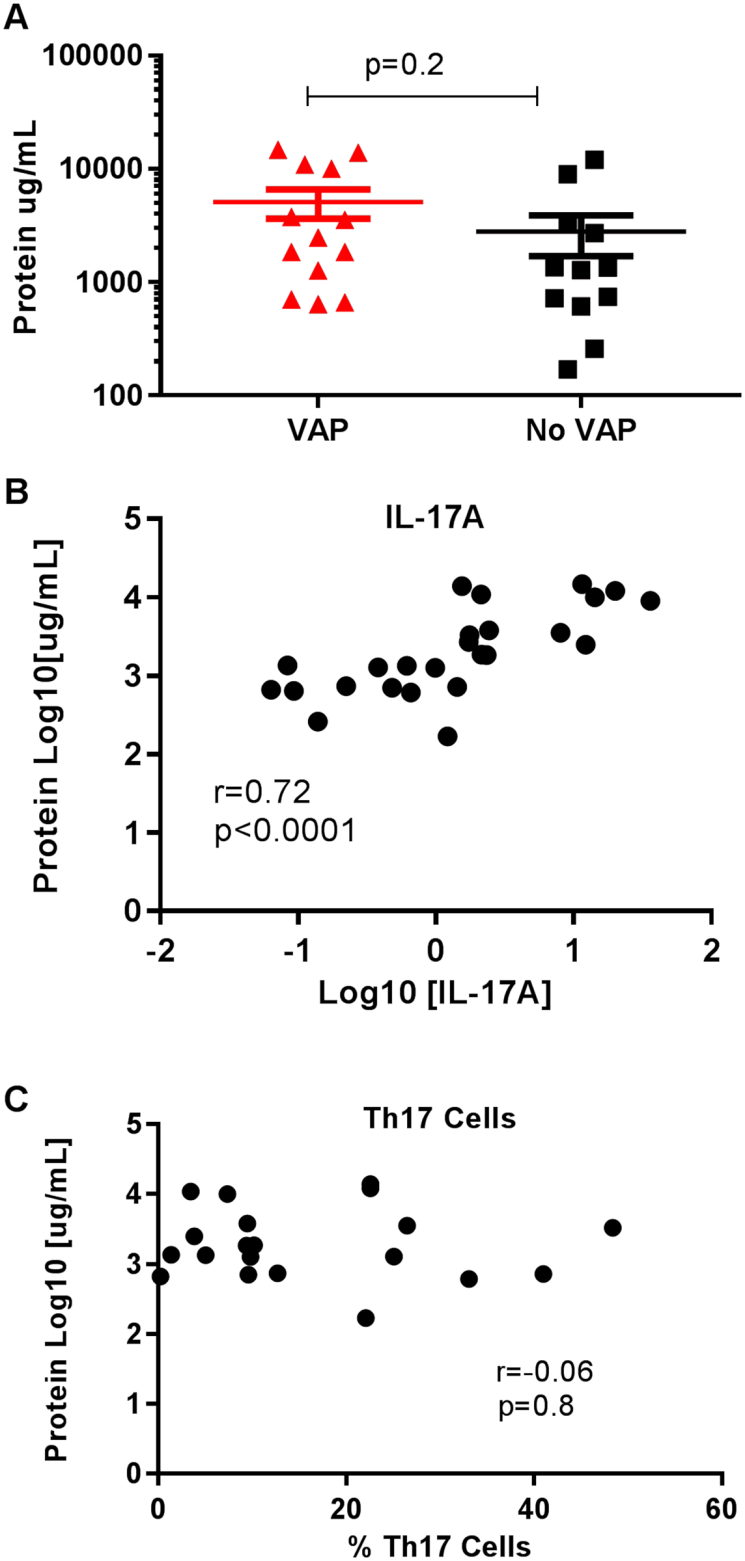
Increased protein in bronchoalveolar lavage correlates to levels of IL-17A protein. Total protein level was measured in BAL using an ELISA. A. There is no significant difference in the amount of protein between the VAP and no VAP groups (p = 0.2). B. Correlation between log of IL-17A cytokine concentration (x-axis) and log of protein concentration (y = axis). C. Correlation between percentage of Th17 cells (x-axis) and log of protein concentration (y-axis).

We next quantified total protein concentration in the BAL fluid as a surrogate for measuring alveolar leak. There was no difference in the amount of protein in the BAL in the VAP vs No VAP group (p = 0.2, Fig 4A). Total protein levels correlated significantly and positively with IL-17A protein levels (r = 0.72, p<0.001, Fig 4B) but not Th17 cell numbers (r = -0.06, p = 0.8, Fig 4C).

**Fig 5:**
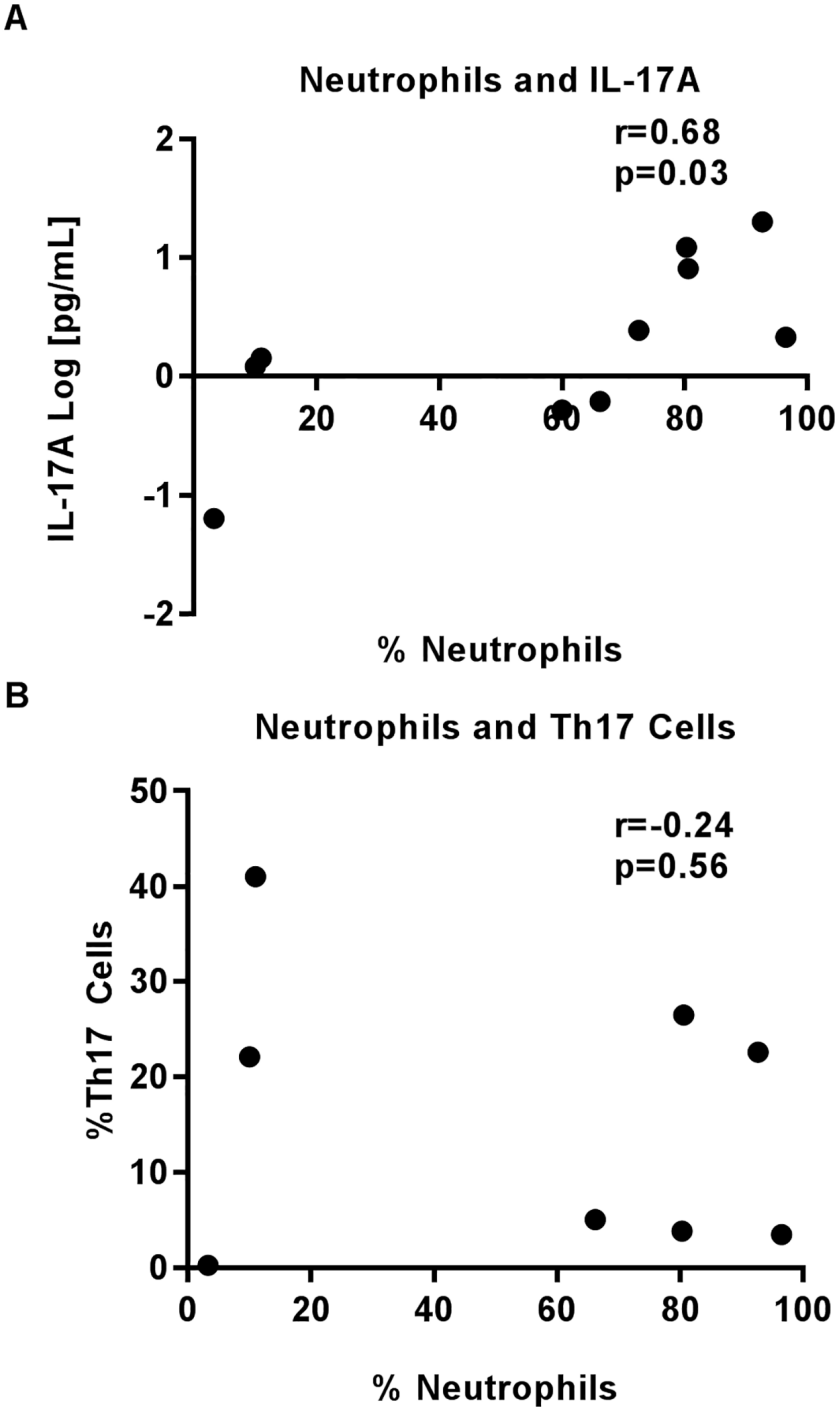
Neutrophil numbers in BAL correlate to IL-17A protein levels. Cytospins were counted for total numbers of neutrophils present in each BAL sample. Correlation between percentage of neutrophils (x-axis) and A) IL-17A protein concentration (log, y-axis) and B) percentage of Th17 cell numbers.

Lastly, we were interested in seeing if neutrophil numbers were better correlated with IL-17A protein levels or Th17 cell numbers. Neutrophil cell numbers correlated significantly and positively with IL-17A protein levels (r = 0.68, p = 0.03, Fig 5A) but not Th17 cell numbers (r = -0.24, p = 0.56, Fig 5B).

## Discussion

It has been demonstrated that Th17 cells and IL-17A are protective against certain pulmonary infections in murine models of pneumonia but the role for these factors in determining the risk for nosocomial pulmonary infections in humans is unclear. Here, we show for the first time that even though there is an increased pro-inflammatory cytokine response in the lungs of humans in the setting of VAP, consistent with previous studies [16,21], the percentage of Th17 cells are lower in patients with ventilator associated pneumonia compared to those without. Levels of CD4+ IFN-γ producing cells were no different between the two groups suggesting that activated CD4+ cells are not preferentially lost, but that the decrease is specific to the Th17 compartment. These data suggest that Th17 cells may be protective against ventilator associated pneumonias in humans.

Several reports in the literature also suggest that Th17 cells are potentially protective against lung infections in humans. For example, patients with Hyper-IgE syndrome lack functional Th17 cells and are at increased risk for pulmonary infections [22]. HIV patients with chronic disease have a preferential depletion of Th17 cells putting them at increased risk for opportunistic infections [23]. In hospitalized patients with community acquired pneumonia alveolar Th17 cells are increased compared to healthy controls suggesting Th17 cells are part of a typical host response to bacterial pneumonia [16]. In contrast, our results show that patients with VAP have a decreased percentage of Th17 cells. Our finding is consistent with the notion of immune paralysis in response to critical illness in the setting of sepsis [3] and suggests that better characterization of post-critical illness immunosuppression is warranted.

We also show, for the first time, that in the setting of VAP the percentage of Th17 cells do not correlate to levels of IL-17A protein and that there is likely an alternate source of IL-17A. Similar reports exist in the oncology literature demonstrating a disconnect between Th17 cell numbers and IL-17A protein levels. In a meta-analysis examining outcomes from all cancers types, the authors conclude that higher levels of Th17 cells are associated with a better prognosis while higher levels of IL-17A are associated with a poor prognosis [24]. Most literature that exists for pulmonary infections assumes that if there is an increased level of IL-17A protein there are an increased number of Th17 cells responsible for secreting the measured IL-17A. Our data suggest that this may be a false assumption. There is a robust literature showing alternative cell types capable of secreting IL-17A including innate lymphoid cells, neutrophils, NK T-cells, and γδ-T cells [5]. Elucidating the source of IL-17A will enhance our understanding of its role in bacterial pneumonia.

In this study, we show that increasing IL-17A levels correlate with an increased percentage of alveolar neutrophils and total alveolar protein concentration. One of the main mechanisms of IL-17A in controlling bacterial pneumonia in mouse models is neutrophil recruitment [5]. In mouse models of lung injury, IL-17A has also been shown to play a role in increased alveolar leak [9]. Taken together, these findings suggest that while there are several putative roles for IL-17A in pulmonary infection, that these mechanisms are likely independent of Th17 cell function in ventilator associated pneumonia.

There are a number of limitations to our study. The first is that we had a small sample size of only 25 patients. Nonetheless, consistent with previously reported literature from our institution, there was a high proportion of VAP amongst patients undergoing bronchoscopy allowing us to compare two evenly distributed groups [25]. Second, our samples were predominantly from Caucasian males at an academic medical center which could make our results less generalizable. Third, there was likely some variability in the timing from onset of VAP to time of alveolar sampling. At our institution, the diagnostic standard is that BAL typically occurs within hours of clinical suspicion. Fourth, in order to avoid batch effects during flow cytometry, we performed experiments using frozen samples. This may introduced the possibility of differential viability, though there was no difference in the number of CD4+ IFN-γ producing cells between the two groups suggesting that there was likely no difference in activated cell viability during the processing steps. Lastly, lymphocytes are a relatively rare cell population in the BAL samples. To enrich for this cell population we positively select CD4 cells in order to separate them from mostly alveolar macrophages and neutrophils, though it is unlikely that positive selection would change our cell populations in any way between the two groups.

In summary, we show that Th17 cell numbers are reduced in patients with VAP even in the setting of a pro-inflammatory cytokine environment. We also show that, as previously reported, IL-17A is likely responsible for increased alveolar leak and neutrophil recruitment, yet the source of IL-17A protein in ventilator associated pneumonia is currently unknown. The surprising finding that Th17 cells and IL-17A protein levels do not correlate suggests that Th17 cells are exhibiting a protective effect through a currently unrecognized mechanism that deserves further investigation.

## Supporting information

S1 Dataset Individual subject experimental data. Proportions of Th17 and IFN-ɣ cells amongst the CD4 population and bronchoalveolar lavage cytokine concentrations.
(XLSX)

S1 Table Individual subject data. Subject demographic, ventilator, and quantitative culture data.
(DOCX)
