# Supplementary material for: Th17 cells are associated with protection from ventilator associated pneumonia
Source: PLoS One. 2017 Aug 14;12(8):e0182966. doi: 10.1371/journal.pone.0182966 (PMC5555641; doi:10.1371/journal.pone.0182966)
Supplement: S1 Table — Subject demographic, ventilator, and quantitative culture data. (DOCX) [file pone.0182966.s002.docx]

S1 Table: Individual Participant Data

|  | Age | Gender | Race/Ethnicity | VAP | APACHE III | Admit Diagnosis | Quantitative Culture Results (CFUs/ml) | P:F Ratio | ICU day | Ventilation day |
| --- | --- | --- | --- | --- | --- | --- | --- | --- | --- | --- |
| 1 | 63 | Male | Caucasian | No | 90 | Sepsis |  | 182 | 4 | 4 |
| 2 | 59 | Male | Caucasian | Yes | 49 | Trauma | 40,000 *Nisseria species*  7,000 *Seratia marcescens* | 134 | 3 | 3 |
| 3 | 58 | Male | Caucasian | Yes | 63 | Sepsis | 1,000 *Enterobacter cloacae*  3,000 *Staphylococcus aureus* (MSSA) | 150 | 13 | 12 |
| 4 | 59 | Male | AA | No | 99 | IPH |  | 337 | 3 | 3 |
| 5 | 31 | Male | Unknown | Yes | 74 | Trauma | 10,000 *Staphylococcus aureus* (MRSA) | 227 | 5 | 5 |
| 6 | 61 | Male | Caucasian | No |  | Trauma |  | 335 | 8 | 8 |
| 7 | 28 | Male | Caucasian | Yes | 69 | Trauma | >10,000 *Haemophilus influenzae*  20,000 *Streptococcus* | 246 | 3 | 3 |
| 8 | 61 | Male | Caucasian | No | 44 | Sepsis |  |  | >28 |  |
| 9 | 29 | Female | Caucasian | No | 25 | Trauma |  | 350 | 8 | 8 |
| 10 | 61 | Male | Caucasian | No | 69 | Trauma |  |  | 15 | 15 |
| 11 | 58 | Male | Caucasian | Yes | 40 | Trauma | >2,000 *Klebsiella oxytoca* on protected specimen brush (PSB)  2,500 *Enterobacter cloacae* | 178 | 15 | 15 |
| 12 | 66 | Male | Caucasian | Yes | 85 | MI | >10,000 *Haemophilus influenzae*  5,000 *Streptococcus*  3,000 *Staphylococcus aureus* (MSSA) | 212 | 10 | 10 |
| 13 | 59 | Male | Caucasian | Yes | 44 | Trauma | 40,000 *Niserria* species  7,000 *Serratia* | 134 | 3 | 3 |
| 14 | 57 | Male | Caucasian | No |  | Sepsis |  | 315 | 3 | 3 |
| 15 | 21 | Male | Caucasian | No | 56 | Trauma |  | 218 | 4 | 4 |
| 16 | 59 | Male | Alaskan Native | Yes | 79 | Trauma | 200 *Enterococcus faecalis* | 220 | 9 | 9 |
| 17 | 27 | Male | Caucasian | Yes |  | Trauma | >10,000 *Pseudamonas* | 198 |  | 23 |
| 18 | 55 | Male | Caucasian | Yes | 28 | Stroke | 60,000 *Streptococcus viridan*s  200 *Haemophilus influenzae* | 176 | 2 | 1 |
| 19 | 41 | Male | Alaskan Native | Yes | 55 | IPH | >100,000 *Staphylococcus aureus* (MRSA) | 272 | 7 | 7 |
| 20 | 68 | Male | Caucasian | Yes | 93 | Trauma | 9,000 *Klebsiela oxytoca* | 178 | 25 | 25 |
| 21 | 53 | Male | Caucasian | Yes | 53 | Trauma | 50,000 *Staphylococcus aureus* (MSSA) | 343 | 14 | 14 |
| 22 | 57 | Male | Caucasian | No | 46 | Sepsis |  | 410 | 1 |  |
| 23 | 65 | Male | Caucasian | No | 72 | Sepsis |  | 523 | 16 | 6 |
| 24 | 22 | Male | Hispanic | No | 82 | Sepsis |  | 472 | 1 | 1 |
| 25 | 28 | Male | AA | No | 47 | Trauma |  |  | 6 | 6 |

AA: African American, IPH: Intraparenchymal Hemorrhage, MI: myocardial infarction, CFUs: Colony forming units, PSB: Protected specimen brush, P:F ratio: PaO_2_:FiO_2_ ratio

Number of ICU/ventilation days prior to bronchoscopy
